# GSK-3β-mediated regulation of cadmium-induced cell death and survival

**DOI:** 10.1186/s11658-018-0076-2

**Published:** 2018-03-12

**Authors:** Seungwoo Kim, Hyosoon Cheon, Sam-Moon Kim, Young-Youl Kim

**Affiliations:** 1 Division of Brain Diseases, Center for Biomedical Science, National Institute of Health, Center for Disease Control & Prevention, Osong Health Technology Administration Complex, 187, Osongsaengmyeong2-ro, Osong-eup, Heungdeok-gu, Cheongju-si, South Korea; 2 Division of Biobank for Health Sciences, Center for Genome Science, National Institute of Health, Center for Disease Control & Prevention, 200 Osongsaengmyeong2-ro, Osong-eup, Heungdeok-gu, Cheongju-si, South Korea

**Keywords:** Cadmium, ER-stress, GSK-3β

## Abstract

**Background:**

Previous studies indicated that cadmium (Cd) increases PI3-kinase/Akt phosphorylation, resulting in an alteration in GSK-3β activity. However, the mechanism of Cd-induced endoplasmic reticulum (ER) stress in neuronal cells has yet to be studied in needs further elucidation. We examined the role of GSK-3β in Cd-induced neuronal cell death and the related downstream signaling pathways.

**Methods:**

SH-SY5Y human neuroblastoma cells were treated with 10 or 20 μM BAPTA-AM and 1 μM wortmannin for 30 min and then incubated with 25 μM Cd for 12 h. Apoptotic cells were visualized via DAPI and PI staining. Data were evaluated with one-way analysis of variance (ANOVA) followed by Student’s *t*-test. Data are expressed as the means ± SD of experiments performed at least three times.

**Results:**

Treatment of human neuronal SH-SY5Y cells with Cd induced ER, stress as evidenced by the increased expression of GRP78, which is a marker of ER stress. Cd exposure significantly increased the phosphorylation of Akt at thr308 and ser473 and that of GSK-3β at ser9 in a time-dependent manner, while the total protein levels of GSK-3β and Akt did not change. Cd-induced apoptosis was higher in GSK-3β-knockdown cells than in normal cells.

**Conclusions:**

Our data suggest that Akt/GSK-3β signaling activated by Cd is involved in neuronal cell survival.

## Introduction

Cadmium (Cd) is a potent toxic metal that affects various cellular processes, such as cell proliferation and apoptosis. It can cause DNA damage, reactive oxygen species (ROS) production and endoplasmic reticulum (ER) stress [1, 2]. The latter two events are important triggers of the stress response in many cell types [1–3].

In neuronal cells, Cd activates c-Jun N-terminal kinase (JNK) or p38 through ROS production, resulting in apoptosis [3–6]. However, the signaling pathways involved in Cd-induced ER stress and apoptosis in neurons are poorly understood.

Previously, we found that Cd induces neuronal cell death through ROS production activated by GADD153. The exposure of SH-SY5Y cells to Cd led to an increase in intracellular GADD153 and Bak levels in dose- and time-dependent manners [7].

The ER regulates protein synthesis, folding and tracking [8], and is highly sensitive to alterations in calcium homeostasis and perturbations of its intralumenal environment. Calcium ionophores, ER Ca^2+^-ATPase inhibitors (e.g., thapsigargin), protein glycosylation inhibitors (e.g., tunicamycin) and misfolded proteins can all disrupt ER function. In response to such ER stresses, protective signal transduction mechanisms such as the induction of glucose-regulated protein 78 (GRP78) [6, 7] or molecular chaperones of the heat shock protein (Hsp70) family are activated. Alternatively, an apoptotic pathway may be initiated, via the activation of transcription factor GADD 153/CHOP or the ER-resident cysteine protease, caspase-12 [7, 9, 10]. Thus, ER stress has been linked to neurodegenerative diseases such as cerebral ischemia [11] and Alzheimer’s disease [12], which involve neuronal apoptosis. Autopsy studies suggest that the PERK-EIF2α pathway is hyperactive in the brains of patients with Alzheimer’s disease [13], implying the presence of ER stress.

Glycogen synthase kinase-3 (GSK-3) is a ubiquitously expressed serine/threonine kinase that regulates glycogen synthesis and controls multiple cell signaling pathways [14, 15]. Two mammalian GSK-3 isoforms, GSK-3α (51 kDa) and GSK-3β (47 kDa), are highly similar and share substrate specificity in vitro, but are encoded by different genes [16]. GSK-3β is a constitutive active kinase that regulates many intracellular signaling pathways by phosphorylating substrates such as β-catenin [17], cAMP response element-binding protein (CREB) [18] and tau [19] in SH-SY5Y cells. GSK-3β activity is regulated through its phosphorylation by other protein kinases, including Akt. For example, in response to insulin and growth factor stimulation, GSK-3β activity is negatively regulated by phosphorylation at serine 9 (Ser9) by the survival-promoting kinase Akt, which is activated in response to various mitogens and growth factors. Other phosphorylation sites are threonine 390 and thyrosine 216 in GSK-3β, the activities of which are respectively negatively and positively regulated [20, 21].

There is evidence that Cd increases PI3-kinase/Akt phosphorylation [22], resulting in an alteration in GSK-3β activity. However, the mechanism of Cd-induced ER stress in neuronal cells has yet to be studied in needs further elucidation. Here, we investigated the role of intracellular calcium and Cd-induced ER stress in the regulation of GSK-3β activity.

## Materials and methods

### Materials

Dulbecco’s modified Eagle’s medium (DMEM), fetal bovine serum (FBS) and antibiotics were purchased from Gibco BRL. Cadmium chloride (Cd), Nonidet-P40 (NP-40), wortmannin (PI3K inhibitor), thapsigargin (Ca^2+^-ATPase inhibitor) and N-acetyl-L-cysteine (NAC) were obtained from Sigma. [1,2-bis(2-aminophenoxy)ethane-*N,N,N′,N′*-tetraacetic acid tetrakis (acetoxy methyl ester)] BAPTA-AM (a Ca^2+^ Chelator) was obtained from Calbiochem-Novabiochem. GSK-3β antibodies were obtained from Cell Signaling Technology. GRP78, GRP94 and GADD153 were obtained from BD Biosciences.

### SH-SY5Y cell culture

SH-SY5Y human neuroblastoma cells (ATCC CRL-2266) were cultured in DMEM supplemented with 10% FBS, 100 U/ml penicillin, 100 mg/ml streptomycin and 0.25 mg/ml amphotericin B. Cells were maintained at 37 °C in a humidified atmosphere of 95% air/5% CO_2_.

### Western blot analysis

Cells were lysed in RIPA buffer consisting of 50 mM Tris-HCl (pH 7.5), 1% NP-40, 0.5% sodium deoxycholic acid and 0.1% SDS with proteinase inhibitors: 1 mM phenylmethylsulfonyl fluoride, 2 μg/ml aprotinin and 2 μg/ml leupeptin. Cellular debris was removed by centrifugation at 12,000×*g* for 20 min. An aliquot (40 μg) of the total protein was separated using 12.5% SDS-PAGE, and the proteins were transferred onto a nitrocellulose membrane (Amersham Bioscience). After the membrane was blocked with 5% fat-free milk in TBST buffer consisting of 25 mM Tris-HCl (pH 7.4), 137 mM NaCl, 5 mM KCl and 0.2% Tween 20 for 1 h, it was incubated with the appropriate primary and secondary antibodies for 1 h each and then developed using an enhanced chemiluminescence kit from Amersham Bioscience. All antibodies were used at a dilution of 1:1000.

### Calcium measurement

Cells were treated with 25 μM Cd for the indicated times, then washed with phosphate-buffered saline (PBS), harvested using trypsin, suspended in PBS, and incubated with 4 μM fluo-4 at 37 °C for 1 h. The cells were centrifuged at 1000×*g* for 5 min, washed three times with PBS, resuspended in PBS, and incubated at 37 °C for 20 min. Intracellular Ca^2+^ was analyzed using flow cytometry (FACSCanto; BD Biosciences).

### DAPI staining and annexin V assay

Cells were treated with 10 or 20 μM BAPTA-AM for 30 min and then incubated with 25 μM Cd for 12 h. The cells were washed with PBS, fixed for 30 min with 4% paraformaldehyde (PFA) prepared in PBS, treated with RNase (1 mg/ml), and incubated with 4′,6-diamindine-2-phenilindole (DAPI; 10 μg/ml) for 30 min. Apoptotic cells with condensed or fragmented nuclei were visualized under a fluorescence microscope. Approximately 100–200 cells per well were assessed. Apoptotic cells were detected by annexin V binding using a kit from Molecular Probes, Inc. according to the manufacturer’s instructions.

### Cell staining with propidium iodide

Cells were treated with 1 μM wortmannin for 30 min and then incubated with 25 μM Cd for 12 h. The cells were washed with PBS, harvested using trypsin and stained with propidium iodide (PI; 5 μg/ml). The cells were analyzed using flow cytometry (FACSCanto; BD Biosciences) and the extent of apoptosis was determined based on the sub-G_1_ population.

### Statistical analysis

Data were evaluated with one-way analysis of variance (ANOVA) followed by Student’s *t*-test. The data are expressed as the means ± SD of experiments performed at least three times. Statistically significant differences are reported as *p < 0.05, **p < 0.01 or ***p < 0.001. Data with values of *p* < 0.05 were generally accepted as statistically significant.

## Results

### Cd induces neuronal cell apoptosis through ER stress

Cd induces cell death in many cell types [22]. To assess Cd-induced apoptosis in human neuronal cells, SH-SY5Y cells were treated with 25 μM Cd for 0, 6, 12, 18 and 24 h. Nuclear staining with the fluorescent dye DAPI were revealed chromatin condensation and fragmentation in the Cd-treated cells (data not shown).

**Fig. 1:**
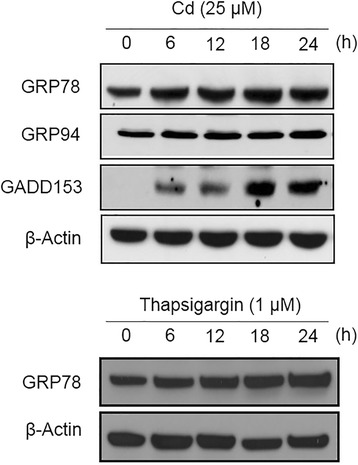
Cadmium induces ER stress in human neuronal cells. SH-SY5Y cells were treated with 25 μM Cd or1 μM thapsigargin for the indicated times. Total cellular proteins were extracted and separated using SDS-PAGE. Western blot analysis was performed using anti-GRP78, anti-GRP94 and anti-GADD153 antibodies

To determine whether Cd induces ER stress in SH-SY5Y cells, we examined the protein expression levels of three ER stress markers, GRP78, GRP94 and GADD153, after Cd treatment. The expression levels of GRP78 and GADD153 had increased and this increase was sustained for up to 24 h after Cd treatment (Fig. 1). The expression of GRP94 and β-actin had not changed following Cd treatment. Cells treated with thapsigargin (TG; 1 μM for 6 ~ 24 h) as a positive control for ER stress showed an increase in the cellular GRP78 protein level after 12 h (Fig. 1). These results are consistent with earlier results [3] suggesting that Cd induces not only ROS but also ER stress in neuronal cells.

**Fig. 2:**
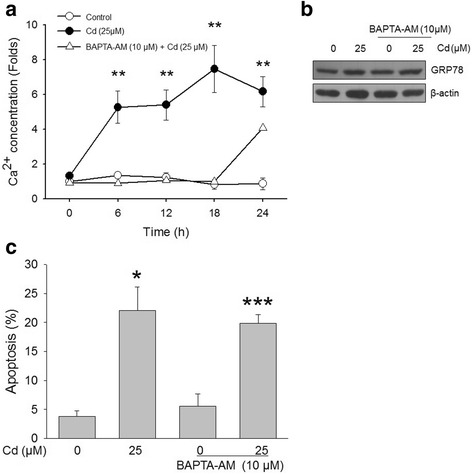
Cadmium increases intracellular Ca^2+^ concentration in neuronal cells. SH-SY5Y cells were treated with or without 10 μM BAPTA-AM for 30 min and then incubated with 25 μM Cd for the indicated times. **a** – Ca^2+^ concentration was quantified using the calcium indicator dye, Fluo-4 AM. **b** – GRP78 was analyzed via western blotting after 30 min pretreatment with 10 μM BAPTA-AM, followed by 12 h incubation with 25 μM Cd. **c** – Apoptotic cells were counted using the annexin V assay. The data are expressed as the means ± SEM of the apoptotic cells from at least three independent experiments. *p < 0.05, **p < 0.01, ***p < 0.001

The observable increase in cytoplasmic Ca^2+^ results from either an influx from the extracellular environment or efflux from intracellular ER stores, and is associated with the initiation of apoptosis in diverse in vivo and in vitro systems [23]. To investigate the role of intracellular Ca^2+^ in Cd-induced ER stress, Ca^2+^ was measured over time via flow cytometry using the calcium indicator dye fluo-4 (Fig. 2a). Intracellular Ca^2+^ was significantly elevated after Cd treatment, increasing as early as 0.5 h after exposure. It oscillated, but reached a peak of around 8-fold higher than the baseline 18 h after Cd exposure. However, the increase in intracellular Ca^2+^ was blocked by pretreatment with 10 μM BAPTA-AM, a Ca^2+^ chelator. To examine the increase in intracellular Ca^2+^ associated with ER stress, the GRP78 protein level was assessed in cells pretreated with BAPTA-AM. As shown in Fig. 2b, Cd-induced upregulation of GRP78 was not inhibited by the Ca^2+^ chelator. In comparison, the apoptosis assay using annexin V were revealed that the pretreatment of cells with 10 μM BAPTA-AM did not protect cells significantly from Cd-mediated apoptosis (Fig. 2c). This result indicates that ER stress, which induces upregulation of GRP78, may play an important role in Cd-induced apoptotic cell death, but that the apoptotic pathway was independent of the intracellular Ca^2+^ level.

### Cd increases GSK-3β phosphorylation via Akt

**Fig. 3:**
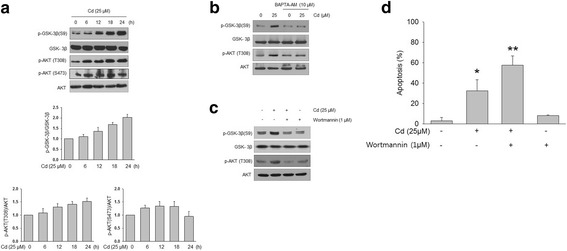
Akt and GSK-3β regulation by cadmium. SH-SY5Y cells were treated with 25 μM Cd for the indicated times. Total cellular proteins were extracted and separated using SDS-PAGE. **a** – Western blot analysis was performed using anti-phospho-Akt (T308), S473 and phospho-GSK-3β (S9) antibodies. Band intensities were quantified based on densitometric values using Fujifilm Science Lab 97 Image Gauge software (version 2.54). The data are from at least three independent experiments. **b**, **c** – p-AKT and p-GSK-3β were analyzed via western blotting after 30 min pretreatment with 10 μM BAPTA-AM (**b**) or 1 μM wortmannin (**c**), followed by 12 h incubation with 25 μM Cd. **d** – Apoptotic cells were counted using the annexin V assay. The data are expressed as the means ± SEM of the percentage of apoptotic cells from at least three independent experiments. *p < 0.05, **p < 0.01

GSK-3β is a serine/threonine protein kinase known for its role in neuropathological disorders. In response to p90^rsk1^ and MEK1/2, GSK-3β is phosphorylated at ser9 to negatively regulate its activity or at tyrosine 216 (tyr216) to positively regulate its activity [14]. Using an antibody directed against phospho-ser9, we examined whether Cd induced GSK-3β phosphorylation at ser9 in SH-SY5Y cells. Figure 3a shows that the phosphorylation of Akt at thr308 and ser473 and that of GSK-3β at ser9 were significantly increased by exposure to 25 μM Cd in a time-dependent manner, while the total protein levels of GSK-3β and Akt did not change. Thus, Cd negatively regulates GSK-3β activity and positively regulates Akt activity.

When cells were pretreated for 30 min with the Ca^2+^ chelator BAPTA-AM (10 μM) before treatment with 25 μM Cd, the Cd-induced upregulation of Akt and GSK-3β phosphorylation was found to have significantly decreased (Fig. 3b). When Akt activation was inhibited by wortmannin, the phosphorylation levels of both Akt (at thr308) and GSK-3β (at ser9) were significantly lower than those found when was used Cd alone (Fig. 3c). These results suggest that Cd inactivates GSK-3β through Akt activation. When neuronal cells were treated with wortmannin for 30 min before treatment with 25 μM Cd for 12 h, the number of apoptotic cells was significantly higher than for cells with no pretreatment (Fig. 3d). These findings indicate that the Akt/GSK-3β pathway plays a survival role in Cd-induced cell death in SH-SY5Y cells.

### Knockdown of GSK-3β increases cd-induced apoptosis

**Fig. 4:**
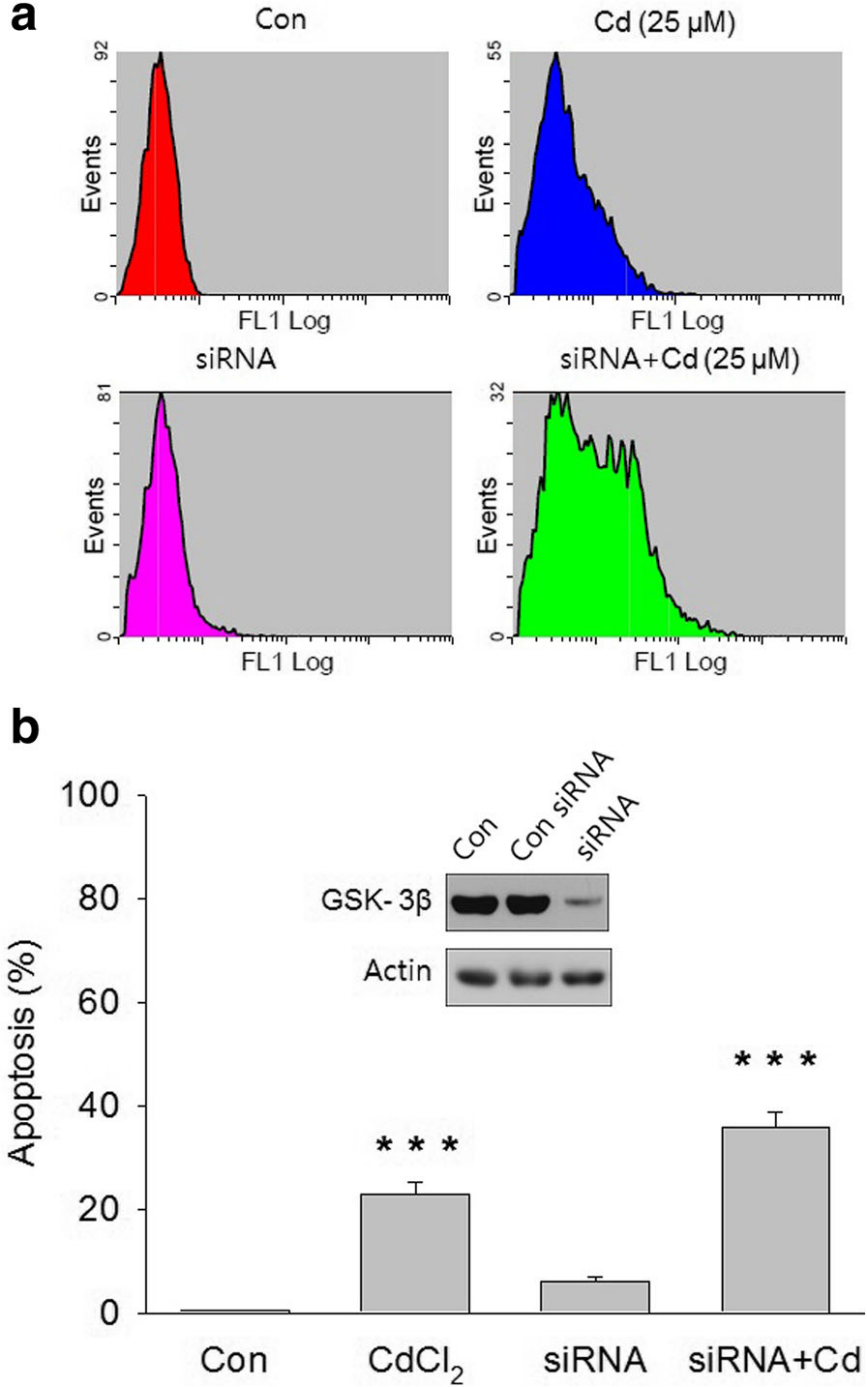
Cadmium-induced apoptosis was increased by GSK-3β knockdown. SH-SY5Y cells were transfected with GSK-3β siRNA, and total proteins were extracted and separated using SDS-PAGE. **a** – Representative FACS data for apoptosis are shown. **b** – Western blot analysis was performed using anti-GSK-3β antibodies (inset). Apoptotic cells were counted using the annexin V assay. The data are expressed as the means ± SEM of the percentage of apoptotic cells from at least three independent experiments. ***p < 0.001

Several studies have proposed that GSK-3β activation results in apoptosis. Although Cd induced increases in the phosphorylation of Akt and GSK-3β (Fig. 3a), the role of GSK-3β in Cd-induced apoptosis in SH-SY5Y cells was unclear. To investigate this, cells were transfected with small interfering RNA (siRNA) targeted against the GSK-3β coding region and then treated with Cd. Cd-induced apoptosis was increased in GSK-3β-knockdown cells compared to the level for normal cells (Fig. 4a and b). These results suggest that GSK-3β is likely to be a signal leading to cell death.

## Discussion and conclusions

Owing to its central role in the induction of multiple signaling pathways, Ca^2+^ is crucial for the sustainability of biological functions, including learning and memory, fertilization, proliferation, development and cell death [24]. Li et al. reported that Cd is a potent Ca^2+^ channel blocker that inhibits cellular Ca^2+^ uptake [25]. By contrast, Cd also increases intracellular Ca^2+^ by inhibiting Ca^2+^-ATPase in the ER membrane. In addition, Cd activates some calcium-related enzymes such as protein kinase C [26], mitogen-activated protein kinase [27] and calmodulin-dependent kinase [28]. Furthermore, the elevation of intracellular Ca^2+^ has been reported to disrupt mitochondrial Ca^2+^ equilibrium, resulting in the formation of ROS [29].

Recent studies have shown that an increase in intracellular Ca^2+^ attributable to Ca^2+^ release from the ER leads to the activation of calpain proteases and caspase-12, which leads in turn to cell death [25, 30]. Our previous data showed that SH-SY5Y cells with NAC showed reduced nuclear fragmentation and condensation and caspase activation [3]. NAC did not affect the regulation of GRP78 or GSK-3β. However, BAPTA-AM significantly inhibited the activity of GSK-3β, demonstrating that Cd-induced ROS generation was not required for ER stress and that GSK-3β played a central role in the regulation of apoptosis. This discrepancy indicates marked differences in the regulation of stress responses (i.e., oxidative stress due to ROS vs. ER stress) depending on the cell type and stimulus.

**Fig. 5:**
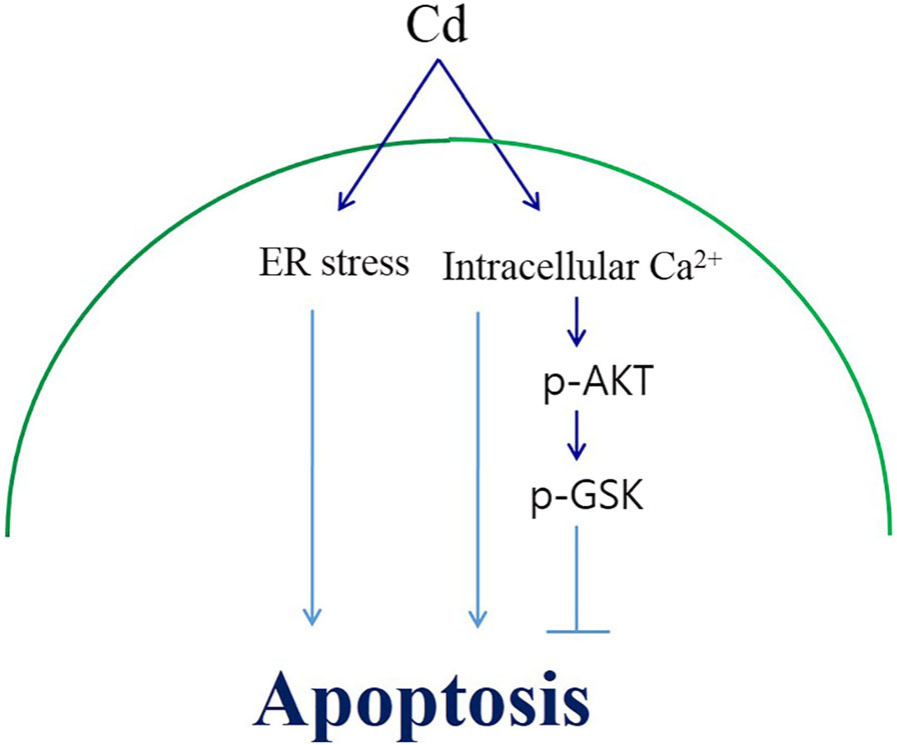
Scheme of the proposed pathways mediating calcium-induced apoptosis. Cd induces ER stress that triggers the pro-apoptotic pathway, including the CHOP and JNK pathways. By contrast, Cd also causes elevation of intracellular Ca^2+^ that contributes to the anti-apoptotic signal via phosphorylation of Akt/GSK3β, and counteracts pro-apoptotic signals

GSK-3β is key node in modulating substrate recognition and kinase activity, which is inhibited by pro-survival PI3K-AKT signaling. Activated Akt plays a survival role in many cell types [31], whereas GSK-3β has a death signaling role after induction by other extracellular stimuli [32, 33]. In previous studies, Cd has been shown to increase Akt phosphorylation [34–36]. In this study, Cd increased Akt and GSK-3β phosphorylation, and an Akt inhibitor, wortmannin, decreased both Akt and GSK-3β phosphorylation (Fig. 3). Thus, Cd decreased GSK-3β activity through increased Akt phosphorylation. In addition, Cd-induced apoptosis was increased by wortmannin pretreatment and GSK-3β siRNA. These results suggest that the role of GSK-3β is not apoptosis but that the survival signal in Cd induces an increase in intracellular Ca^2+^ (Fig. 5).

The pro-survival activity of GSK-3 through NF-kB has been shown to play a role in chronic lymphocytic leukemia (CLL) [37]. GSK-3 activity also contributes to pro-survival NF-kB signaling through the phosphorylation of the NF-kB inhibitory protein p100. GSK-3β is crucially involved in many signaling pathways. Its role in the regulation of apoptosis is not constant, and in some situations GSK-3β promotes cell survival [38].

In early ER stress, translational attenuation occurs to reduce the ER load. During the next phase, several groups of genes are transcriptionally induced for long-term adaptation to ER stress. If severe ER stress conditions persist, apoptosis signaling pathways are activated, including induction of CHOP and activation of JNK and caspase-12. Thus, the influence of ER stress on cell death and survival depends on the balance between apoptosis and survival [39].

These results provide evidence that Cd-induced apoptosis of neuronal cells is mediated, at least in part, by ER stress, and that regulation via a distinct GSK-3β pathway is implicated in the apoptotic process. As demonstrated in this study, Cd can produce both pro- and anti-apoptosis signals, although under the conditions of our experiments, Cd treatment ultimately resulted in apoptosis.

## Abbreviations

Cd: Cadmium
ER: Endoplasmic reticulum
GRP78: Glucose-regulated protein78
GSK-3β: Glycogen synthase kinase-3 β
